# The Relation Between Arterial Hypertension and Cognitive Impairment: A Literature Review

**DOI:** 10.7759/cureus.52782

**Published:** 2024-01-23

**Authors:** Gabriel Zúñiga Salazar, Diego Zúñiga, Sneha Balasubramanian, Khawar Tariq Mehmood, Shahad Al-Baldawi

**Affiliations:** 1 Medicine, Universidad Católica de Santiago de Guayaquil, Guayaquil, ECU; 2 Internal Medicine, Madras Medical College, Chennai, IND; 3 Internal Medicine, Aster Hospital Br of Aster DM Healthcare FZC, Dubai, ARE; 4 Rheumatology, Al-Yarmouk Teaching Hospital, Baghdad, IRQ

**Keywords:** cognitive decline, mild cognitive impairment, cognitive impairment, dementia, arterial hypertension, hypertension

## Abstract

The global increase in dementia cases, driven by improved life expectancy and reduced elderly mortality rates, presents a significant public health challenge. Dementia, characterized by a gradual and irreversible decline in cognitive abilities, affects individuals aged 65 and older, disrupting lives and straining healthcare systems. Hypertension significantly influences dementia development. Research consistently links midlife hypertension to cognitive decline, mild cognitive impairment (MCI), and dementia, but findings in older adults vary. While some studies suggest that late-life hypertension accelerates cognitive decline and dementia risk, others propose a protective effect. The impact of hypertension on cognition varies across age groups, spanning from childhood to late life. High blood pressure during midlife and earlier life stages consistently predicts poorer cognitive outcomes. Executive function, attention, and motor speed are the cognitive domains most affected by hypertension, particularly in subcortical diseases. Memory impairments in hypertension-related dementias are complex, often overlapping with other causes.

Understanding the inconsistent findings in older adults regarding hypertension, cognitive decline, and dementia risk requires comprehensive exploration of methodological and biological factors. Addressing hypertension and its management may hold the key to reducing the risk of cognitive decline and dementia, especially in midlife and earlier life stages.

## Introduction and background

Mortality rates in the elderly have gone down, and life expectancy has risen. As a result, there's been a global increase in dementia cases. Dementia is a syndrome that typically affects individuals aged 65 and older, marked by a gradual and irreversible decline in cognitive abilities, including attention, memory, executive function, visuospatial skills, and language [1]. It disrupts the lives of those affected and their families, posing a significant public health concern.

Dementia is a prominent contributor to both mortality and disability. According to the World Health Organization [2], approximately 55.2 million individuals were affected by dementia across the globe in 2019. Projections suggest that this number will grow to 78 million by 2030 and 139 million by 2050. The 2020 Lancet Commission on Dementia indicated that 40% of dementia cases could potentially be averted or postponed by addressing the risk factors linked to it [3].

Vascular risk factors, such as high blood pressure (BP), have a significant influence on the development of dementia because as many as 50% of patients diagnosed with Alzheimer's disease (AD) exhibit mixed pathology upon autopsy, which includes cerebrovascular damage [4]. Additionally, it's widely accepted that having hypertension in midlife raises the risk of developing dementia in later life, regardless of a person's genetic predisposition for dementia [5]. Consequently, there's a continued need to understand the mechanisms that link hypertension and dementia, making it a top research priority.

While research has hinted at the potential of reducing risks through effective BP management [6], the results have been varied and inconclusive [7]. Additionally, the debate about whether specific categories of anti-hypertensive medications offer superior cognitive advantages remains unsettled [8].

In this review, we look into how hypertension can lead to cognitive decline and dementia and assess their practical implications.

## Review

Methods

A MEDLINE (PubMed) literature review was performed by using the following search queries: (Cognitive decline AND Hypertension) OR (Cognitive decline AND Blood pressure) OR (Cognitive impairment AND Hypertension) OR (Cognitive impairment AND Blood pressure) OR (Dementia AND Hypertension) OR (Dementia AND Blood pressure).

**Figure 1:**
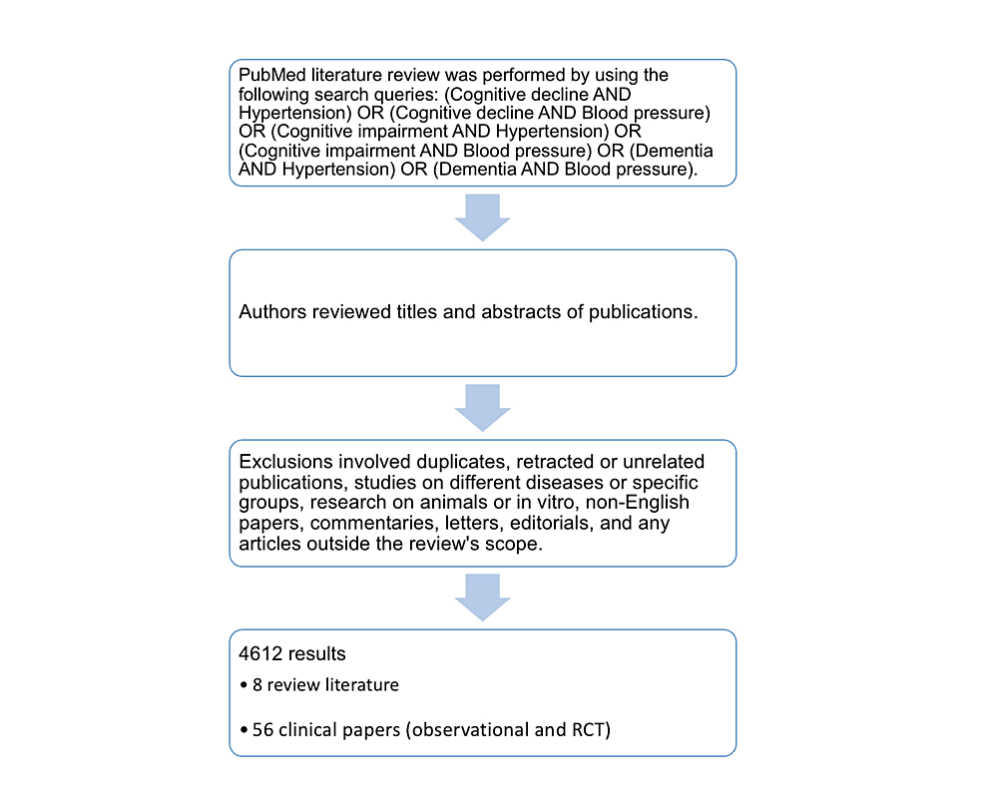
Methods and selection

The authors reviewed titles and abstracts of publications. Exclusions involved duplicates, retracted or unrelated publications, studies on different diseases or specific groups, research on animals or in vitro, non-English papers, commentaries, letters, editorials, and any articles outside the review's scope. From 4612 results, 64 publications meeting the specified criteria were chosen (Figure 1).

Impact of hypertension on cognition

An increasing amount of evidence underscores hypertension as a risk factor for negative cognitive consequences. This evidence is predominantly derived from epidemiological research that identifies hypertension in community settings as a risk factor for cognitive decline, mild cognitive impairment (MCI), and dementia [9]. For definitions of these three different entities, refer to Table 1.

**Table 1:** Definitions of cognitive decline, mild cognitive impairment, and dementia Credits: Gabriel Zúñiga Salazar

Terminology	Definition
Cognitive decline	Gradual reduction in cognitive abilities with age, affecting memory, thinking, and reasoning.
Mild cognitive impairment	Notable cognitive changes beyond age-related norms but not severely interfering with daily life, potentially a transitional stage.
Dementia	Severe cognitive decline disrupting daily activities, stemming from diverse causes like Alzheimer's disease, vascular issues, or Lewy body disease.

Research focused on cognitive decline enables the assessment of alterations in cognitive function, thereby detecting even minor instances of cognitive impairment, without the necessity of meeting explicit dementia or MCI criteria. Additionally, it provides a less convoluted method of gauging cognitive well-being, facilitating a more unadulterated evaluation of the link between hypertension and cognitive abilities [10].

On the other hand, research that focuses on MCI and dementia as endpoints carries a more compelling public health message because these terms are clearer and more readily understood. Preventable disease cases are more readily acknowledged in comparison to alterations in the steepness of cognitive decline trajectories [10,11].

Regarding hypertension, epidemiological studies provide substantial evidence for several outcomes: hypertension is unequivocally linked to accelerated cognitive decline [11,12], subpar cognitive abilities [13], and the occurrence of new cases of MCI and dementia [14-17]. Moreover, in the case of adults diagnosed with MCI, high BP is linked to a heightened likelihood of cognitive deterioration and a decline in cognitive function [18].

What was previously categorized as prehypertension, defined as a systolic blood pressure (SBP) less than 140 mmHg, has been identified as a potential risk factor for dementia in various studies. This includes the Atherosclerosis Risk in Communities (ARIC) cohort study [19], where both midlife hypertension and prehypertension were associated with approximately 40% higher dementia risk compared to individuals with normal BP.

Research based on population data has consistently demonstrated that, spanning from midlife through the geriatric phase, there is a clear association between hypertension and the development of future dementia, including both AD and vascular dementia (VaD) [20-30] (Table 2).

**Table 2:** Studies that showed a relationship/correlation between hypertension and cognitive impairment SBP: systolic blood pressure; DBP: diastolic blood pressure; BP: blood pressure; PP: pulse pressure; BPV: blood pressure variability; HTN: hypertension; CASI: Cognitive Assessment Abilities Screening Instrument; IQCODE: Informant Questionnaire on Cognitive Decline in the Elderly; MMSE: Mini-Mental State Examination; HDS: Hasegawa Dementia Scale; MoCA: Montreal Cognitive Assessment; CDR: Clinical Dementia Rating; CERAD-NB: Consortium to Establish a Registry for Alzheimer's Disease Neuropsychological Battery; DSST: Digit Symbol Substitution Test

Author	Study design	Number of cases	BP parameter	Outcome measure	Result
Launer et al. [20]	Prospective	3703 normal population	SBP, DBP	CASI, IQCODE	BP↑: risk for dementia ↑ in drug-naïve men
Kivipelto et al. [21]	Prospective	1449 normal population	SBP, DBP	MMSE	SBP↑: risk for dementia ↑
Yamada et al. [22]	Retrospective	No dementia: 1660; dementia: 114	SBP	CASI, MMSE, HDS	SBP↑: risk for dementia ↑
Whitmer et al. [23]	Prospective	8845 normal population	Diagnosis of HTN	Diagnosis of Dementia	Hypertension: risk for dementia ↑
Yoshitake et al. [24]	Prospective	828 normal population	SBP	MMSE, HDS	SBP↑: risk for dementia ↑
Qin et. al. [25]	Prospective	277 MCI patients	Diagnosis of HTN	MMSE, MoCA, CDR	Hypertension: risk for dementia ↑
Zúñiga-Salazar et al. [26]	Cross-section	Hypertensive, non-demented 60	SBP, DBP, diagnosis of HTN	MoCA	Hypertension duration↑: MoCA score ↓; SBP↑: MoCA score ↓
Bahchevanov et al. [27]	Cross-section	No dementia: 112	Diagnosis of HTN	CERAD-NB	Hypertension: CERAD-NB score ↓
Boo et al. [28]	Prospective	4289 normal population	BP	MMSE	BP↑: risk for dementia ↑
Sun et al. [29]	Prospective	1369 normal population	SBP, DBP, PP	Verbal Learning Test, DSST, Stroop Interference Test	10 mmHg ↑ in SBP, DBP, PP: DSST score ↓
Shim and Shin [30]	Cross-section	Impaired cognition: 174	SBP, short-term BPV	MMSE	SBP↑: risk for dementia ↑

In a prospective study with a population of 3703 people by Launer et al., an increase of dementia cases was observed in patients with high BP [20]. Yamada et al. found, in a retrospective study with 1449 people, that an increase in SBP led to an increase in dementia in this population [22]. A cross-sectional study with 174 patients with cognitive dysfunction by Shim and Shin found that there was an increase in the SBP of these individuals [30]. A cross-sectional study by Zúñiga-Salazar et al. shows that hypertension is correlated with MCI [26]. In a comprehensive analysis that encompassed 17 distinct systematic reviews, it was concluded that hypertension increases the risk of VaD and cognitive decline, although the link to AD is less pronounced [31]. However, some other studies have proposed either that there is no connection between hypertension and dementia [32,33] or that the relationship is the opposite, suggesting a protective effect [34]. Furthermore, the connection between BP and cognition may exhibit variations depending on the age group [35-37].

Cognitive domains affected

Extensive neuropsychological assessments conclude that hypertension's most significant impact is on executive function [11], motor speed, and attention [38]. These domains are traditionally associated with subcortical diseases, such as typical vascular disease or pure VaD [39]. Even scores on global cognitive tests like the Mini-Mental State Examination (MMSE) and Montreal Cognitive Assessment (MoCA), although lower in individuals with more vascular risk factors, including hypertension, are primarily influenced by difficulties related to attention (in the case of the MMSE) and visuo-executive function (in individuals with lower MoCA scores) [40].

Memory impairments in hypertension-related dementias are complex due to potential overlaps in causes and the high prevalence of mixed dementias. While memory problems are typically more associated with cognitive impairments in AD compared to VaD, individuals with both hypertension and elevated brain amyloid, as detected by Pittsburgh compound B positron emission tomography (PiB-PET) scans, have shown memory impairments [41].

Nonetheless, many of these studies focusing on specific cognitive domains are influenced by misdiagnoses or difficulties in determining the exact causes during an individual's lifetime. When analyzing neuropathological data, it was observed that cognitive domains did not significantly differ based on etiology as might be expected: individuals with pure AD neuropathology had reduced memory scores, especially compared to executive function, while those with cerebrovascular disease showed similarly reduced cognitive function in domains related to executive function and verbal and nonverbal memory [42].

Pathophysiology 

Cognitive impairment is influenced by several factors related to hypertension. One key factor is atrophy and brain damage resulting from both macro- and microinfarcts and hemorrhages. Even relatively small volumes of damage in specific brain regions responsible for cognition can lead to cognitive dysfunction [43]. Additionally, microinfarcts can have a more significant functional impact than expected based on their size [44]. The extent and location of white matter lesions are also linked to the severity and progression of cognitive dysfunction [45]. These lesions can impede cognitive function by disrupting the connectivity between the anterior thalamus and the frontal cortex [45].

**Figure 2:**
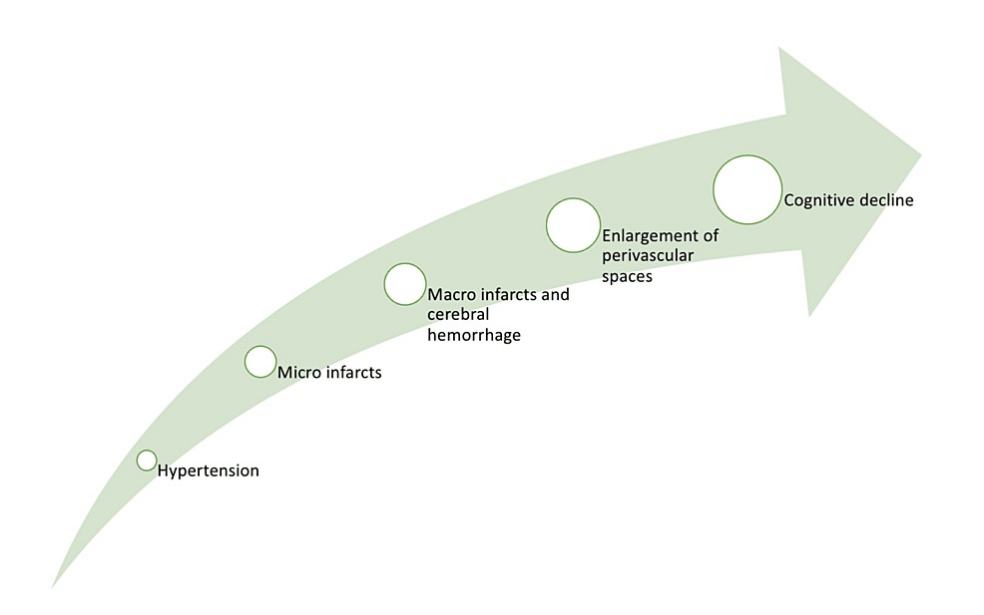
Pathophysiology of cognitive decline due to hypertension

The effect of enlarged perivascular spaces on hypertension-related cognitive impairment is not yet fully understood. Changes in the structure of perivascular spaces suggest that the clearance systems around blood vessels may be affected, potentially contributing to white matter damage [46]. In hypertension, perivascular spaces are both enlarged and distorted, which could hinder the removal of potentially harmful byproducts of brain activity (Figure 2).

Age relationship between BP and cognitive impairment

The data consistently show that having high BP during midlife (ages 45-64) is a risk factor for late-life cognitive decline [11], cognitive impairment [37], and dementia [11], regardless of an individual's initial cognitive abilities in midlife. Adults with hypertension who take anti-hypertensive medications experience a decelerated cognitive decline compared to those with untreated hypertension [37]. Moreover, midlife hypertension is linked to an accelerated cognitive decline in midlife [47]. The adverse impact of elevated BP on cognitive function may initiate during childhood (ages 10-18) and young adulthood (ages 18-44) [48-50]. For instance, individuals with higher BP levels from young adulthood to midlife exhibit poorer cognitive performance in midlife [51]. Elevated SBP in young adulthood is associated with the development of young-onset dementia [52]. This cumulative research suggests that the detrimental effect of BP on cognitive decline and dementia might span an individual's entire life.

Nevertheless, the connection between elevated BP and dementia risk in older adults (aged 65 and above) exhibits varying outcomes. Certain long-term studies indicate that higher SBP in older adults is correlated with faster cognitive decline and an increased risk of dementia, potentially with more pronounced effects as age advances [53,54]. Conversely, additional observational studies involving individuals have reported that late-life hypertension is associated with decelerated cognitive decline and reduced dementia risk [55].

A study from the United Kingdom [56] examining BP and dementia/vascular cognitive impairment (VCI) yielded diverse findings in two different cohorts. In the Clinical Practice Research Datalink dataset, the link between elevated SBP and a heightened risk of VaD diminished with age [56]. Nevertheless, higher SBP predicted a higher risk of all-cause dementia over a five-year period, with no indication of an adverse association in older age within the Oxford Vascular Study data [56]. 

To summarize, the evidence indicates that elevated BP during childhood, young adulthood, and midlife is linked to poorer cognitive outcomes. Corresponding with this body of research, some studies have revealed that elevated SBP in late life serves as a risk factor for faster cognitive decline and an increased risk of dementia [20]. Nevertheless, other investigations have suggested that the detrimental impact of high BP on cognitive decline and dementia diminishes with increasing age or that high BP may even have a protective effect against dementia risk.

Understanding the reasons for these inconsistent findings in older adults regarding BP, cognitive decline, and dementia risk requires a more comprehensive exploration of methodological and biological factors influencing the outcomes.

Anti-hypertensive therapy for dementia prevention

Given robust evidence linking hypertension and cognitive function, numerous authors propose that reducing SBP may decrease the risk of cognitive impairment and dementia [3].

Several groundbreaking randomized controlled trials (RCTs), spanning from the 1990s to the present, prioritized stroke, major adverse cardiovascular events, and death as primary outcomes. An early intervention study, the Medical Research Council's hypertension trial, found no cognitive difference between active and placebo-treated groups, concluding that hypertension treatment caused no cognitive harms [57].

Studies such as the Systolic Hypertension in the Elderly Program (SHEP) [58], Hypertension in the Very Elderly Trial (HYVET) (mean age, 83.5 years) [59], Heart Outcomes Prevention Evaluation-3 (HOPE-3) [60], and Study on Cognition and Prognosis in the Elderly (SCOPE) [61], excluding those with prior stroke or dementia, revealed no significant cognitive outcome differences between active and control treatment groups.

In the Epidemiology of Vascular Aging (EVA) study group, treating hypertension was associated with less cognitive decline over a four-year period compared to untreated hypertension [62]. 

The Systolic Hypertension in Europe (Syst-Eur) trial demonstrated that dementia-free individuals aged ≥60 years with hypertension (SBP 160-219 mmHg and DBP <95 mmHg), who received medication to lower BP to <150 mmHg systolic, showed less incident dementia over a median follow-up of two years, as opposed to those on a placebo [63].

The combined evidence from the Systolic Blood Pressure Intervention Trial (SPRINT) and Systolic Blood Pressure Intervention Trial-Memory and Cognition in Decreased Hypertension (SPRINT-MIND) provided definitive clinical trial proof that aggressive BP control in individuals with high cardiovascular risk reduced incident MCI and combined MCI and dementia, excluding those with a history of stroke [64].

Finally, in various observational studies, the use of anti-hypertensive medication is linked to reduced cognitive decline. According to the ARIC study, participants on anti-hypertensive medications experienced a 20-year cognitive decline comparable to a prehypertensive group, which was higher than normotensives but lower than untreated hypertensives [11]. 

## Conclusions

The relationship between hypertension and cognitive function is complex and multifaceted. While there is substantial evidence linking hypertension to cognitive decline, MCI, and dementia, the impact of hypertension on cognition varies across different age groups. High BP during midlife and earlier stages of life appears to be a consistent risk factor for cognitive impairment and dementia in later life. However, in older adults, the relationship between late-life hypertension and cognitive decline is less clear, with some studies suggesting potential protective effects. The cognitive domains most affected by hypertension include executive function, attention, and motor speed, which are associated with subcortical diseases and vascular risk factors. Memory impairments in hypertension-related dementias are complex and can overlap with other causes, making it challenging to pinpoint specific cognitive deficits based on etiology. To better understand the discrepancies in older adults, further research is needed to explore the methodological and biological factors that influence these outcomes. Overall, addressing hypertension and its management may play a crucial role in reducing the risk of cognitive decline and dementia, especially in midlife and earlier stages of life.
